# Insight into sleep quality and its relationship with emotional intelligence: results of a cross-sectional study among Italian university students

**DOI:** 10.3389/fpubh.2024.1392571

**Published:** 2024-05-15

**Authors:** Francesca Licata, Riccardo Maruca, Emma Antonia Citrino, Aida Bianco

**Affiliations:** ^1^Department of Health Sciences, School of Medicine, University of Catanzaro “Magna Græcia”, Catanzaro, Italy; ^2^Department of Medical and Surgical Sciences, School of Medicine, University of Catanzaro “Magna Græcia”, Catanzaro, Italy

**Keywords:** emotional intelligence, healthy lifestyle, Italy, sleep quality, university students

## Abstract

**Background:**

This study aimed to investigate sleep habits and examine the relationship between sleep quality and its potential predictors, namely Emotional Intelligence (EI) and perceived health status.

**Methods:**

The present cross-sectional study was conducted between February 13 and February 28, 2023, at the “Magna Græcia” University of Catanzaro, in the Southern part of Italy. The study involved undergraduate students who were 18 years or older, proficient in the Italian language, and with no restrictions on the major attended. They completed a self-administered survey on socio-demographic information, health status, sleep quality (Pittsburgh Sleep Quality Index-PSQI), EI, and perceived health status.

**Results:**

The majority of the sample (59.6%) was enrolled in medical or life science majors. The results showed a high prevalence of poor sleep quality and sleep latency was identified as the most affected aspect of it. The female gender and the self-perceived health status were the strongest predictors of poor sleep quality. Poor sleepers showed lower emotional clarity, emotional repair, and total EI scores. Moreover, as age increased, the odds of being classified as a poor sleeper increased by 7%.

**Conclusion:**

The survey highlights that poor sleep health is still a significant issue and empowering individuals to make proactive decisions to adopt healthy lifestyles in the early phase of life is of paramount importance. The study exhibited the interesting role of EI in influencing sleep quality, highlighting that when emotional events are insufficiently regulated, it may result in sleep disturbances. Therefore, the promotion of sleep quality requires an integrated yet innovative approach including emotion regulation.

## Introduction

Along with food, water, and air, sleep is a biological necessity for human life. It's vital for the health and wellbeing of children, adolescents, and adults. In a contemporary 24-h society, poor sleep health is a pervasive problem and represents an under recognized public health challenge strongly associated with critical health outcomes, including cardiovascular diseases, obesity, mental health problems, and neurodegenerative diseases (1). According to international recommendations, adults should sleep at least 7 h per night and experience good sleep quality to avoid physical and mental health issues (2). In the United States, only 65% of adults report sleeping the recommended 7 h or more per day leaving one-third of the population at risk of sleep deficiency (3). Similar data were found in Italy (4). There is a widespread misperception that sleep issues are only an epiphenomenon of other disorders as a result of the significant overlap with other mental and physical health morbidities (5). However, evidence demonstrates that sleep problems can play a causal role in the development of these conditions (6).

The assumption that sleep quality is merely adversely oriented toward disease rather than positively oriented toward health is challenged by the recently developed idea of “sleep health” (1). This concept recognizes the multidimensionality of sleep, which occurs at the individual level and in a larger socio-ecological context. In accordance with this conceptualization, studies showed that loneliness and social isolation are associated with poorer sleep health indicators (7) and that the quality of social and romantic relationships (8), or workplace interactions (9) may affect sleep health. Furthermore, research has shown that even little smartphone and laptop usage (i.e., 1 h of blue light exposure), can modify melatonin secretion by 25%, acting as potential risk factors for developing sleep disturbances (10). Exposure to light at night may disturb biological, behavioral, and physiological cycles and induce chrono-disruption (11). Sleep deprivation influences affective processes, which are crucial for personality and social interaction. This may link to emotional intelligence (EI) skills, which enable individuals to accurately identify and interpret their emotions and others', and regulate their behavior in social contexts (12). Research shows that individuals with higher EI levels have better sleep health (13), suggesting that understanding and managing emotions may promote optimal sleep patterns and contribute to the adoption of healthy lifestyles, making EI a health-oriented skill (14).

University students are particularly exposed to the risk of developing sleep problems. The tendency to go to bed late at night, combined with an intense nocturnal social life, increased consumption of alcohol and coffee, non-optimal living conditions (e.g., room-sharing), and the need to follow university schedules, are risk factors for cumulative sleep loss and poor sleep quality (15). Previous research has demonstrated that sleep quality is associated with the experience of positive emotion, and also with reported engagement in strategies to regulate it (16). Moreover, sleep quality appears to be linked with both emotion intensity and regulation. Evidence exists that emotion affects sleep, as well as sleep regulates emotion (17). Emotional events during daytime and adaptive emotion regulation affect sleep. Emotional response is influenced by sleep quality in a reciprocal way.

Hence, considering EI abilities and sleep habits closely related to each other and their interconnectedness ultimately impacting general wellbeing (18), we speculated that higher levels of EI among university students could be positively associated with better sleep quality. This hypothesis is based on the understanding that individuals with greater EI may possess improved coping mechanisms, stress management skills, and emotional regulation abilities (3), which are conducive to fostering healthy sleep patterns and reducing the likelihood of experiencing poor sleep quality. Therefore, the present study aimed to investigate sleep quality and its relationship with EI and other potential predictors among university students.

## Materials and methods

### Study design and setting

The present cross-sectional study was conducted, during the winter exam session, between February 13th and February 28th, 2023, at the “Magna Græcia” University of Catanzaro, in the Southern part of Italy.

### Study size

The sample size was calculated using a single population proportion formula, with the assumption that there was a 5% margin of error, a 95% confidence level, and a hypothetical 60% prevalence of poor sleepers among university undergraduate students (19). According to this method, a sample of at least 370 university students was required. By anticipating a low response rate, a larger sample of students was invited to participate in the study, and the questionnaire was sent to 740 university students.

### Participant recruitment

The inclusion criteria were: (i) being enrolled as an undergraduate student at the university without the restriction of major attended, (ii) being willing to provide written informed consent, and (iii) being aged 18 and older. Those who refused to give informed consent and did not have good knowledge of the Italian language were excluded from the sample. The randomly selected students received, via institutional email, the link to the self-administered survey, built using a Google Forms^®^ online application. An agreement on personal data treatment was requested as a mandatory step to start filling out the Google form. Participants were guaranteed confidentiality by using coding systems and secure data storage methods. A summary of the study objectives was also supplied on the front page of the questionnaire. In order to prevent duplicate survey responses, the questionnaire could only be completed once by each respondent, limiting to one submission of the IP addresses. Participants did not perceive any form of payment or incentive for taking part in this study.

### Questionnaire design

The survey was divided into four sections exploring: (1) socio-demographic information and health status; (2) sleep quality; (3) the level of EI; (4) and perceived health status.

#### Socio-demographic information and health status

The first section of the questionnaire contained questions related to socio-demographic information, including gender, age, and major attended. Furthermore, each participant was questioned about whether they had any chronic diseases and, if yes, which ones.

#### Sleep quality

Sleep quality was investigated through the Pittsburgh Sleep Quality Index (PSQI), the most commonly self-rated measure used in clinical and research settings. It has shown good validity and reliability in university student samples (20). The tool assesses seven clinical domains of sleep difficulties (i.e., subjective sleep quality, sleep latency, sleep duration, habitual sleep efficiency, sleep disturbances, use of sleeping medications, and daytime dysfunction). The PSQI consists of 19 items, of which 15 are rated on a scale of 0–3 and four are open-ended and recoded to a scale of 0–3. Instructions on how to complete the questionnaire were provided. The higher the score (>5) the lower the sleep quality. The validated Italian version of the instrument was used for this research sample (21).

#### Emotional intelligence

Self-perceived EI was assessed using the Trait Meta-Mood Scale (TMMS). It is a self-administered instrument consisting of 30 items that investigate 3 domains of the emotional experience: emotional attention (EA), which is the tendency to notice emotional states; emotional clarity (EC), which refers to the capacity to identify and differentiate between different emotional states; and emotional repair (ER), which is the ability to regulate emotional states in order to better adapt to situations. The TMMS demonstrated robust psychometric properties and good resistance to cross-cultural adaptations (22). The Italian version of this tool was validated with regards to internal consistency, concurrent validity, and factor structure (22) after a rigorous back translation process carried out by Giovannini et al. (23).

Respondents can answer each item using a Likert-like scale of 5 points, ranging from “Strongly disagree” (1) to “Strongly agree” (5). A score for each domain can be obtained; and the total TMMS score provides a general, composite estimate of the examinee's self-perceived EI.

#### Perceived health status

Following the WHO-recommended standardized question “How is your health in general?”, perceived health status was assessed in the last part of the survey. Participants could rate it using a symmetric scale, with response categories being: “very good, good, fair, poor, very poor.” Perceived health status (PHS) reflects people's overall perception of their health (24). Despite the subjective nature of the question, indicators of perceived health status are a good predictor of people's future use of healthcare and mortality (25).

### Statistical analysis

Means and standard deviations were provided for all normally distributed variables. Medians and the interquartile range (IQR) were used when deviations from normality were encountered. It was possible to estimate the skewness of the variables using Shapiro-Wilk tests. Percentages were used to express categorical variables.

Bivariate analyses were conducted to explore the relationship between sleep quality, EI domains, and total score. *T*-test was performed to determine if there was a significant difference in means if samples were normally distributed; the Wilcoxon-Mann–Whitney test was used if normality was violated. Furthermore, to provide a more accurate analysis, *p*-values have been adjusted using the Bonferroni correction to control for the number of comparisons. Cohen's effect size was also measured to determine the magnitude of the relationship between variables. A logistic regression model was built to explore the role of potential predictors of being classified as poor sleepers (Model 1). In the model, the variables age (continuous, in years), gender (0 = male; 1 = female), majors attended (0 = social science or technology; 1 = medical or life sciences), perceived health status (0 = good/very good; 1 = fair/poor/very poor) and EI (attention, repair, and clarity, all three continuous) were included. If the *p*-value is 0.05 or lower, the result is trumpeted as significant. Adjusted odds ratios (ORs) and 95% confidence intervals (CIs) were calculated. Statistical analysis was developed using Stata Statistical Software, Version 18 (26).

## Results

A summary of the sociodemographic characteristics of participants is provided in Table 1. The sample (*n* = 518) had a median age of 23 years (IQR = 20–25 years) and included 387 female students (74.7**%)**. The sex distribution in the sample mirrors the general trend in the region. More than half (59.6%) were enrolled in medical or life science majors. Of all participants, 355 (68.5%) individuals reported a good or very good perceived health status.

**Table 1:** Sociodemographic characteristics of study participants overall and by sleep quality (*N* = 518).

	**Overall**	**Sleep quality**	
	**% (** ***n*** **)**	**Poor % (** ***n*** **)**	**Good % (** ***n*** **)**
**Sex**			
Male	25.3 (131)	20.5 (61)	31.8 (70)
Female	74.7 (387)	79.5 (237)	68.2 (150)
		χ^2^ = 8.62, 1 df, *p* = 0.003	
**Major**			
Medical or life science	59.6 (309)	58.7 (175)	60.9 (134)
Social science	28.0 (145)	29.5 (88)	25.9 (57)
Technology	12.4 (64)	11.7 (35)	13.4 (29)
		χ^2^ = 0.91, 2 df, *p* = 0.636	
**Perceived health status**			
Good/very good	68.5 (355)	56.0 (167)	85.4 (188)
Fair	22.8 (118)	29.9 (89)	13.2 (29)
Poor/ very poor	8.7 (45)	14.1 (42)	1.4 (3)
		χ^2^ = 55.05, 2 df, *p* < 0.001	

Overall, 57.5% of students (79.5% of the women, and 20.5% of the men) were categorized as “poor sleepers.” The mean sleep duration was 7 h and 8 min, and the mean sleep latency was 29 min. The percentage of students with a sleep latency >30 min was 43.8%. Less than half of the sample (46.1%) had the recommended sleep duration. As shown in Table 2, the PSQI components for sleep latency and sleep disturbances had the highest mean scores (1.40 and 1.33, respectively), and sleep medication and sleep efficiency had the lowest (0.29 and 0.55, respectively). The PSQI subjective sleep quality item had a very good rating in 10.4% (*n* = 54) of individuals, fairly good in 56.4% (*n* = 292), fairly bad in 28 % (*n* = 145), and very bad in 5.2% (*n* = 27). The PSQI sleep medication item indicated that 86.5% (*n* = 448) of participants did not use it during the past month, 2.9% (*n* = 15) had used it less than once a week, 6% (*n* = 31) had used it once or twice a week and 4.6% (*n* = 24) had used it three or more times a week. For more than two-fifths of the sample (43.8%), it was ‘somewhat of a problem' or ‘a very big problem' to have sufficient energy to complete daily activities. Sleep efficiency, defined as the ratio between the time a person spends asleep, and the total time dedicated to sleep, resulted in ≥85% in 63.5% of the sample, between 75% and 84% in 21.6%, between 65% and 74% in 10.8%, <65% in 4.1%. ‘Have bad dreams' and ‘feel too cold' were the most frequently reported trouble sleeping symptoms during the past month, with ‘cannot breathe comfortably' and ‘cough or snore loudly' being the least frequent.

**Table 2:** Descriptive data for Pittsburgh sleep quality index (PSQI).

**Sleep quality (PSQI)**	**Mean (±SD)**	**Range**
Global score	6.58 (±3.40)	0–21
Sleep latency	1.40 (±1.05)	0–3
Daytime disfunction	1.10 (±0.72)	0–3
Sleep disturbances	1.33 (±0.60)	0–3
Subjective sleep quality	1.28 (±0.72)	0–3
Sleep efficiency	0.55 (±0.84)	0–3
Sleep duration	0.63 (±0.67)	0–3
Use of sleep medications	0.29 (±0.78)	0–3

SD, Standard deviation.

Table 3 shows the means and standard deviations of three EI dimensions and total TMMS scores according to sleep quality. EC, ER, and total TMMS scores were significantly lower in poor sleepers than in good sleepers (*p* < 0.001), with a medium effect size (d = 0.59, d = 0.61, and d = 0.59, respectively). No significant differences were found between the EA scores.

**Table 3:** Emotional intelligence univariate analysis according to sleep quality.

	**Poor sleeper**	**Good sleeper**	***p-*value unadjusted**	**Bonferroni adjustment^a^**	**Cohen's d**
	**Mean** ±**SD**	**Mean** ±**SD**			
EA	48.6 ± 7.4	49.4 ± 6.9	0.224	0.198	0.11
EC	34.9 ± 8.1	39.5 ± 7.2	**<** **0.001**	**<** **0.001**	0.59
ER	18.2 ± 4.9	21.1 ± 4.5	**<** **0.001**	**<** **0.001**	0.61
Total TMMS	101.7 ± 14.9	110.0 ± 12.7	**<** **0.001**	**<** **0.001**	0.59

EA, Emotional Attention; EC, Emotional Clarity; ER, Emotional Repair; SD, Standard deviation. Effect size are measured as Cohen's d. ^*a*^Bonferroni correction, *p* < 0.012. Significant values are in bold.

The results of multiple logistic regression analysis (Table 4) demonstrated that female gender (OR: 1.72; 95% CI: 1.10–2.70) and fair, poor, or very poor self-perceived health status (OR: 2.92; 95% CI:1.82–4.68) were the strongest predictors of being categorized as having poor sleep quality. In addition, for each year of age increase (OR: 1.07; 95% Cl: 1.01–1.14) the odds of being classified as a poor sleeper increase by 7%. Finally, poor sleep quality was also positively associated with lower repair (OR: 0.94; 95% CI: 0.89–0.98) and clarity (OR: 0.95; 95% CI: 0.93–0.98) components of EI.

**Table 4:** Results of the regression model for potential determinants of the outcome of interest.

**Outcome: Being categorized with poor sleep quality Log likelihood** = −**305.80009; Prob** > **chi2**<**0.001; Obs** = **518**			
**Variables**	**OR**	**95% CI**	***p*** **-value**
**Perceived health status**			
Good/very good^a^	1.00		
Fair/poor/very poor	2.92	1.82–4.68	<0.001
**Emotional intelligence**			
Clarity, continous	0.95	0.93–0.98	0.001
Repair, continous	0.94	0.89–0.98	0.005
Attention, continous	1.00	0.98–1.02	0.782
**Gender**			
Male^a^	1.00		
Female	1.72	1.10–2.70	0.018
**Age in years, continuous**	**1.07**	**1.01–1.14**	**0.025**
**Majors**			
Social science/Technology^a^	1.00		
Medical or life science	0.96	0.65–1.43	0.862

^a^Reference category.

## Discussion

In light of the high prevalence of poor sleep quality and the serious health consequences it can lead to (1) an augmented understanding of the significance of getting enough sleep is required. The present study offers insights into the potential role of EI and other predictors in sleep quality among university students. Valuable information is provided for healthcare professionals and policymakers to develop and implement effective interventions to improve sleep quality and, ultimately, overall health outcomes. In line with our hypothesis, a high prevalence of poor sleep quality in the sample was shown, confirming that sleep problems represent a relevant issue among university students. Indeed, it is a matter of concern that more than half of the sample fell into the “poor sleeper” category, consistent with previous studies indicating prevalence rates between 50% and 70% (19). However, there might be an overestimation of the result due to the ongoing exam session. Indeed, previous studies reveal significant differences in the amount and quality of sleep that university students experience during the exam period (27–29). For instance, sleep quantity, quality, and daytime functioning decrease from the pre-exam to the exam period but improve from the exam to the post-exam period (28).

Nevertheless, these figures emphasized that poor sleep quality may still be a significant issue during the university career, needing interventions and strategies to promote healthy sleep habits and raise awareness of the negative effects of inadequate sleep on overall wellbeing. Indeed, growing evidence over the past few decades indicates that short sleep duration and poor sleep quality (PSQI > 5) may be linked to unfavorable health outcomes [e.g., increased risk of hypertension (30) and unsatisfactory academic achievements (31)]. In addition, a difference in sleep quality between males and females has arisen. Poor sleep quality showed an increased probability of occurring in female university students. Previous research has shown that as individuals reach pubertal maturity, sex-specific changes in the activity of the hypothalamic-pituitary-adrenal axis occur (32, 33). Despite maintaining almost identical and consistent bedtime and waking hours, women's circadian rhythms for melatonin and body temperature are set to an earlier hour than men's (34). Moreover, the sex differences in sleep disturbances may be explained by higher stress levels and a greater emotional reaction to stressors in female young adults, as stressful life events are a primary risk factor for sleep disturbances in early adulthood (35). Although sleep quality gender-based differences might also be due to socioeconomic pressures and cultural norms, young adults in the study might be less influenced by social pressure than older people (36), and therefore biological factors may be the main cause of gender-based disparities in study participants' sleep quality, as previously demonstrated (37).

Subsequently, the analysis of the PSQI sub-components identified sleep latency as the most affected aspect of sleep quality. Sleep latency is an important dimension of sleep health that is independently associated with hypertension (38) and depressive symptoms (39). Prolonged sleep latency among university students may be attributable to excessive smartphone use, often at bedtime, very common in this population group (40). It is well known that individuals who use smartphones immediately before sleep or view their smartphone screens while trying to sleep may experience reduced sleep quality (41). Electronic devices emit a considerable amount of short-wavelength light (i.e., blue light). Evidence has accumulated that exposure to blue light during the evening can reduce subjective and objective levels of sleepiness and increase alertness. Indeed, it has been demonstrated that smartphone use during the night decreases melatonin production by 1.4 to 15% in dark environments (10) and it can have the potential to impact the circadian rhythm (42). The suppression of melatonin secretion reduces sleep pressure and reinforces the propensity to stay awake (11). It has been demonstrated that, among university students, smartphone users at bedtime had a significantly higher prevalence of increased sleep latency in comparison to non-users (43). Hence, it could be argued that the tendency to delay bedtime that emerged with the present study might happen as a maladaptive coping mechanism that soothes stress and emotional overwhelming: using the smartphone before bed significantly contributes to individuals feeling satisfied and relaxed, further reducing sleep efficiency and increasing sleep latency (44). The potential relationship between dysfunctional emotion regulation and poor sleep hygiene is also corroborated by the significantly lower EC, ER, and total TMMS scores used to assess the level of EI, that have been found in the poor sleeper group compared to the good sleeper ones. Indeed, evidence exists that not being competent in correctly identifying one's emotions and dealing with them, makes people more prone to seeking temporary gratifications (45). This finding expands the range of associations between EI and health-related behaviors (14), providing further evidence about the potential role of EI in reducing unhealthy lifestyles. In light of all the above results, it is plausible to say that a good strategy for tackling poor sleep quality should use both psychological and behavioral interventions.

A further result from the study showed a correlation between an unsatisfactory perception of health status and poor sleep habits. Although the chance of reverse causality cannot be entirely dismissed given the cross-sectional design of the study, this result hinders great potential. Indeed, it is reasonable to argue that perceiving poor health status may be exploited as a key to unlocking better health outcomes. Hence, when individuals perceive their health as poor, they may be more motivated to make necessary lifestyle changes, such as adopting healthier habits or seeking professional medical advice (46). For these reasons, sleep health interventions have to target different groups, such as primary care physicians, who should regularly investigate sleep quality and duration during medical visits. In a study focusing on patient engagement improvement, sleep was found to be the most pertinent aspect of health and quality of life in patients' perceptions, providing further support for the significance of assessing sleep quality (47).

Finally, the figure that the chance of being classified as a poor sleeper increases with age confirms that over the course of natural aging, sleeping habits vary and usually in non-pathological ways (48). Total sleep time is lower in older adults than in younger adults and decreases until approximately the age of 60, after which it stabilizes throughout later life stages. Therefore, empowering individuals to make proactive decisions to adopt healthy lifestyles in the early phase of life is of paramount importance to enhance sleep quality. Prolonged sleep loss may negatively affect emotional development increasing risks for interpersonal conflict as well as more serious mental health problems. Therefore, improving sleep in youth may play a role in preventing and managing these conditions and promoting attention, memory, and analytical thought indispensable to improve academic performances.

### Limits

There are some potential limitations that are worthy of emphasis when interpreting the study results. First, due to the study's cross-sectional design, the findings could be viewed as minimally informative for causal inference and may be susceptible to reverse causality. In particular, reverse causality could pertain to the individual perception of unhealthy status as a potential predictor of poor sleep quality, whereas the relationships could be inverse, i.e., poor sleep health could be independently associated with having a poor self-perceived health status. However, none of these concerns are unique to or inherent in the structure of a cross-sectional study, and assuming that these limitations are materially important risks discarding evidence that may be useful in assessing causal relationships.

Second, as with any survey based on a self-administered questionnaire, information may not be entirely accurate, and the findings reported in this article could be subject to bias. Guarantees were proffered to all respondents that data collection would be protected by confidentiality and anonymity so that they could be confident in answering the question. Moreover, sleep questions could not closely correspond with objective measures of sleep, introducing a reporting bias. Although sleep components were self-reported, in the author's point of view, making information about all dimensions of sleep health available is pivotal for health protection. However, psychometric evaluation of PSQI supports its internal consistency reliability and construct validity, and, in the author's opinion, self-report remains the most practical and cost-effective method for epidemiologic sleep studies involving large population-based samples able to formulate broad prevalence estimates, critical in a context in which no data are yet available.

Third, information regarding the participants' personality traits and lifestyles were not collected in the present study. Although it was outside the current study's scope to evaluate the impact of personality traits on sleep quality, future research should consider including their evaluation to provide more information that can suggest further points of intervention and aid in tailoring prevention and treatment strategies to poor sleepers.

Despite these limitations, this survey serves as an important barometer in understanding pattern of sleep health among university students, highlighting that poor sleep health is still a significant issue that requires attention. Moreover, the innovative contribution of the article lies in its exploration of the potential role of EI and other predictors in influencing sleep quality among university students. Specifically, the correlation between poor sleep quality and lower levels of EI, suggested that individuals who struggle with identifying and regulating their emotions may be more prone to experiencing sleep disturbances. Furthermore, the article suggested the importance of early intervention in promoting healthy sleep habits during adolescence, as sleep patterns tend to vary with age, and prolonged sleep disturbances may become chronic over time.

These data could be useful for healthcare professionals and policymakers to develop targeted interventions aimed at improving sleep quality and, ultimately, overall wellbeing among young adults.

## Data availability statement

The dataset presented in this study can be found in an online repository. The name of the repository and accession number can be found at: Mendeley Data repository (10.17632/nk2ysbyc8z.1).

## Ethics statement

The study involving humans was approved by Calabria Region Local Human Research Ethics Committee (ID no. 36/2023/10/18). The study was conducted in accordance with the local legislation and institutional requirements. The participants provided their written informed consent to participate in this study.

## Author contributions

FL: Writing—original draft, Conceptualization, Data curation, Formal analysis, Validation, Visualization. RM: Writing—original draft, Conceptualization, Data curation, Formal analysis, Validation, Visualization. EAC: Writing—original draft, Conceptualization, Formal analysis, Validation, Visualization. AB: Writing—review & editing, Conceptualization, Formal analysis, Funding acquisition, Investigation, Project administration, Resources, Supervision, Validation.
